# A Randomized Double-Blind Placebo-Controlled Study on the Safety and Efficacy of Probiotics for Insulin Resistance and Oxidative Stress Markers in Diabetic Patients With End-Stage Renal Disease on Hemodialysis

**DOI:** 10.7759/cureus.74621

**Published:** 2024-11-27

**Authors:** Usharani Pingali, Raja Karthik Kalidindi, Padmaja Mekala, Imran Khan

**Affiliations:** 1 Department of Clinical Pharmacology and Therapeutics, Nizam's Institute of Medical Sciences, Hyderabad, IND; 2 Department of Nephrology, Nizam's Institute of Medical Sciences, Hyderabad, IND

**Keywords:** end-stage renal disease, hd (hemodialysis), insulin resistance, oxidative stress, probiotics

## Abstract

Background: Chronic kidney disease (CKD) is a progressive loss of kidney function that can lead to end-stage renal disease (ESRD), requiring renal replacement therapy. Patients on chronic hemodialysis are at a higher risk of developing cardiovascular disease. This study aimed to investigate the effect of 12-week probiotic supplementation on insulin resistance, oxidative stress, and lipid profiles in diabetic patients with ESRD undergoing hemodialysis.

Methods: This is a prospective, randomized, parallel-group, double-blind, placebo-controlled study with efficacy parameters including changes in insulin resistance assessed by homeostatic model assessment for insulin resistance (HOMA-IR); oxidative stress markers malondialdehyde (MDA), nitric oxide (NO), and glutathione (GSH); glycemic control (fasting blood sugar (FBS), glycosylated hemoglobin); and lipid profile over 12 weeks of probiotic supplementation in diabetic patients with ESRD on hemodialysis compared to placebo.

Results: The study included a total of 47 subjects, 30 men and 17 women with a mean age of 52.68 years, and observed a significant reduction in HOMA-IR with a mean difference of 0.66; improvements in MDA, NO, and GSH with mean differences of 0.92, 6.16, and 24.37 µmol/L, respectively; and a significant improvement in FBS and hemoglobin A1c (HbA1c). Minor self-limiting gastrointestinal adverse events like bloating and constipation were associated with probiotics.

Conclusion: Probiotic supplementation can improve insulin resistance in patients with diabetes and ESRD undergoing regular hemodialysis. However, further research is needed to explore its effects on clinical outcomes.

## Introduction

Chronic kidney disease (CKD) has become a major public health challenge worldwide, with a global prevalence of 13.5% [1]. India has more than 115 million CKD patients, according to the Global Burden of Disease Study (2017) [2]. The progression of CKD is usually slow; however, when the patient reaches the most advanced stage of illness, that is, end-stage renal disease (ESRD), kidney function is damaged to the extent that the patient needs renal replacement therapy (RRT), an artificial process used to remove water, electrolytes, and waste substances from the blood. Hemodialysis (HD) is the most used RRT in India [3].

Insulin resistance (IR) and oxidative stress (OS) are common in patients undergoing chronic HD (CHD) and are associated with excess mortality. The etiology of IR and OS in the CHD population is complex and multifactorial. Some of the proposed determinants of IR and OS in CHD patients include chronic inflammation, excess visceral fat, adipokine deregulation and accumulation, metabolic acidosis, OS, vitamin D deficiency, anemia, decreased physical activity, and accumulation of uremic toxins [4]. Loss of vitamins during HD procedure, reduced selenium levels, and reduced function of the glutathione (GSH) scavenging mechanism can further deteriorate OS in HD [5].

Short-chain fatty acids (SCFAs) are aliphatic carboxylic acids of low carbon number (C2-C6) produced by bacterial fermentation of dietary fiber or via protein catabolism, with acetate (C2), propionate (C3), and butyrate (C4) as the main contributors to total SCFA content [6]. SCFAs regulate blood pressure and metabolism by activating G protein-coupled receptors and inhibiting histone acetylation [7].

Probiotics are defined as “live microorganisms which when administered in adequate amounts confer a health benefit on the host” [8]. Although relatively new, the beneficial effects of certain foods containing live bacteria have been recognized for centuries. They are available as capsules, tablets, packets, or powders and are contained in various fermented foods, most commonly in yogurt or dairy drinks. Probiotic products may contain a single microorganism or a mixture of several species [9]. The most widely used probiotics include lactic acid bacteria, specifically *Lactobacillus* and *Bifidobacterium* species. *Saccharomyces boulardii* also appears to have health benefits [10].

*Lactobacillus* (*L.*) spp., *Bifidobacterium* (*B.*) spp., *Streptococcus* spp., *Enterococcus* spp., and *Saccharomyces boulardii* are the most administered probiotic strains for supplementation [11]. By maintaining intestinal epithelial barrier function, competing with pathogens for nutrients, and regulating the host immune response, probiotics can improve host metabolism, relieve uremic toxicity, reduce pro-inflammatory factor levels, and delay the progression of renal injury in CKD patients [12].

In recent years, there has been growing interest in the relationship between gut microbiome and advanced CKD stages. Studies have shown significant diversity in the composition of gut bacteria in patients with advanced CKD and healthy individuals [13]. This suggests that the microbiota composition may be altered during HD treatment, leading to a shift toward more pathogenic bacteria [14].

There are no interventional studies targeted at improving IR in Indian HD patients. The beneficial effects of probiotic supplementation on glycemic control, lipid levels, and biomarkers of OS among non-HD patients have been previously reported [12,13]. The discovery of the gut-kidney axis has created new therapeutic opportunities for a nutritional intervention involving dietary protein, fiber, prebiotic (fibers that are non-digestible food ingredients and beneficially affect the host’s health by selectively stimulating the growth and/or activity of some genera of microorganisms in the colon), probiotic, and synbiotics (combination of prebiotic and probiotic). It has also been reported that the pharmacobiotic potential of the gut microbiota may provide a plausible therapeutic avenue for the administration of live multistrain probiotic cultures.

Following the recent evidence from various animal and human studies of probiotic supplementation in improving antioxidant mechanisms [15], which in turn maintains glucose homeostasis by decreasing IR, this study aims to investigate the effect of multistrain probiotic (*Lactobacilli*, *Bifidobacterium*, and *Bacillus*) supplementation on IR, lipid levels, and biomarkers of OS in the management of diabetic patients with ESRD on maintenance HD.

## Materials and methods

The study protocol was approved by the Institutional Ethics Committee (IEC) and registered in the Clinical Trials Registry, India (CTRI/2021/11/038044). The study was conducted in accordance with the Declaration of Helsinki and Good Clinical Practice Guidelines issued by the Central Drugs Standard Control Organization, the Ministry of Health, and the Government of India. Before each participant was enrolled in the study, a thorough explanation of the study's objective was given, and they voluntarily provided signed informed consent.

Methodology

This was a prospective, randomized, parallel-group, double-blind, placebo-controlled study involving ESRD diabetic patients undergoing HD attending the dialysis unit of the Nephrology Department. Subjects aged 35-65 years of either sex, having type 2 diabetes mellitus (T2DM) for at least five years, who were on stable antidiabetic medication for at least three months prior to the start of the study, with hemoglobin A1c (HbA1c) values in the range of 7%-10% and a homeostatic model assessment for IR (HOMA-IR) value > 4, and with ESRD who were undergoing HD thrice weekly for at least three months prior to the start of the study were included in the study. Pregnant and lactating women, subjects with active gastrointestinal diseases (inflammatory bowel disease, irritable bowel syndrome), subjects who are taking probiotics or have taken probiotics within the last month prior to the start of the study, subjects who were taking antioxidants and/or anti-inflammatory supplements (vitamin E, vitamin C, or omega-3 fatty acids), and subjects who were taking immunosuppressive medication within three months before enrollment in the study were excluded.

Subjects were screened for eligibility during the screening visit, and eligible subjects were randomly assigned to the intervention groups based on a computer-generated randomization list in the allocation ratio of 1:1. The pharmacist supervised treatment concealment. For every randomization number, there was a sealed envelope containing the details of the study medications. All these details were kept confidential and under the custody of the pharmacist. Both the investigator and the subjects were blinded to the treatment allocation. The subjects were assigned to either group A or group B.

Group A received UB0316 capsules, which contained *Lactobacillus salivarius* UBLS-22 (five billion colony-forming units (CFU)), *Lactobacillus casei* UBLC-42 (five billion CFU), *Lactobacillus plantarum* UBLP-40 (five billion CFU), *Lactobacillus acidophilus* UBLA-34 (five billion CFU), *Bifidobacterium breve* UBBr-01 (five billion CFU), *Bacillus coagulans* Unique IS-2 (five billion CFU), and fructooligosaccharide 100 mg. Subjects assigned to group B received a matching placebo, i.e., similar in appearance to probiotic capsules and dispensed in similar containers as probiotics to maintain blinding.

Subjects were instructed to take the study medications once daily 30 min before the afternoon meal for 12 weeks. Follow-up visits were at four, eight, and 12 weeks for assessment of general well-being as well as adverse events and checking compliance to medications using the pill count method. Final measurements and assessments were performed at the end of the treatment period. The safety and tolerability of subjects were monitored throughout the study.

The primary outcome measures include HOMA-IR and the OS markers, malondialdehyde (MDA), nitric oxide (NO), and GSH. HOMA-IR is a calculated parameter derived from serum fasting insulin and serum fasting plasma glucose, calculated as given below:

HOMA-IR = [fasting insulin (µIU/mL)] × [fasting glucose (mg/dL)]/405 [16]

The secondary outcome measures were glycemic control parameters (fasting blood sugar (FBS), HbA1c, and serum fasting insulin) and lipid profile (total cholesterol (TC), high-density lipoprotein cholesterol (HDL-C), low-density lipoprotein cholesterol (LDL-C), triglyceride (TG), and very-low-density lipoprotein cholesterol (VLDL-C)). Safety assessment was based on the incidence and severity of adverse events in both groups.

Statistical analysis

The sample size was calculated based on a study conducted by Soleimani et al. [17]. A sample size of 50 subjects was found to be sufficient to detect a mean difference of 2.6 with a standard deviation (SD) of 3.3 in HOMA-IR, considering a dropout rate of 15% and a screen failure rate of 10%. A total of 62 subjects were needed for the study. The power of the study was 80%. Type I error was set at 0.05. Statistical analysis was performed using GraphPad Prism version 9.3.1 (Insight Venture Management, LLC, New York, NY, US) and Microsoft Office 365 Excel version 2303 (Build 16227.20280) (Microsoft Corp., Redmond, WA, US). All subjects who completed 12 weeks of treatment were considered for efficacy analysis. Data are presented as the mean ± standard deviation for normally distributed data. Comparisons within the group at various time points were performed using a paired t-test. Comparisons between groups were performed using an unpaired t-test. Data from the interrupted patients was not included in the efficacy analysis. All subjects who were randomized in the study were considered for safety analysis. Safety data were expressed as proportions.

## Results

**Figure 1:**
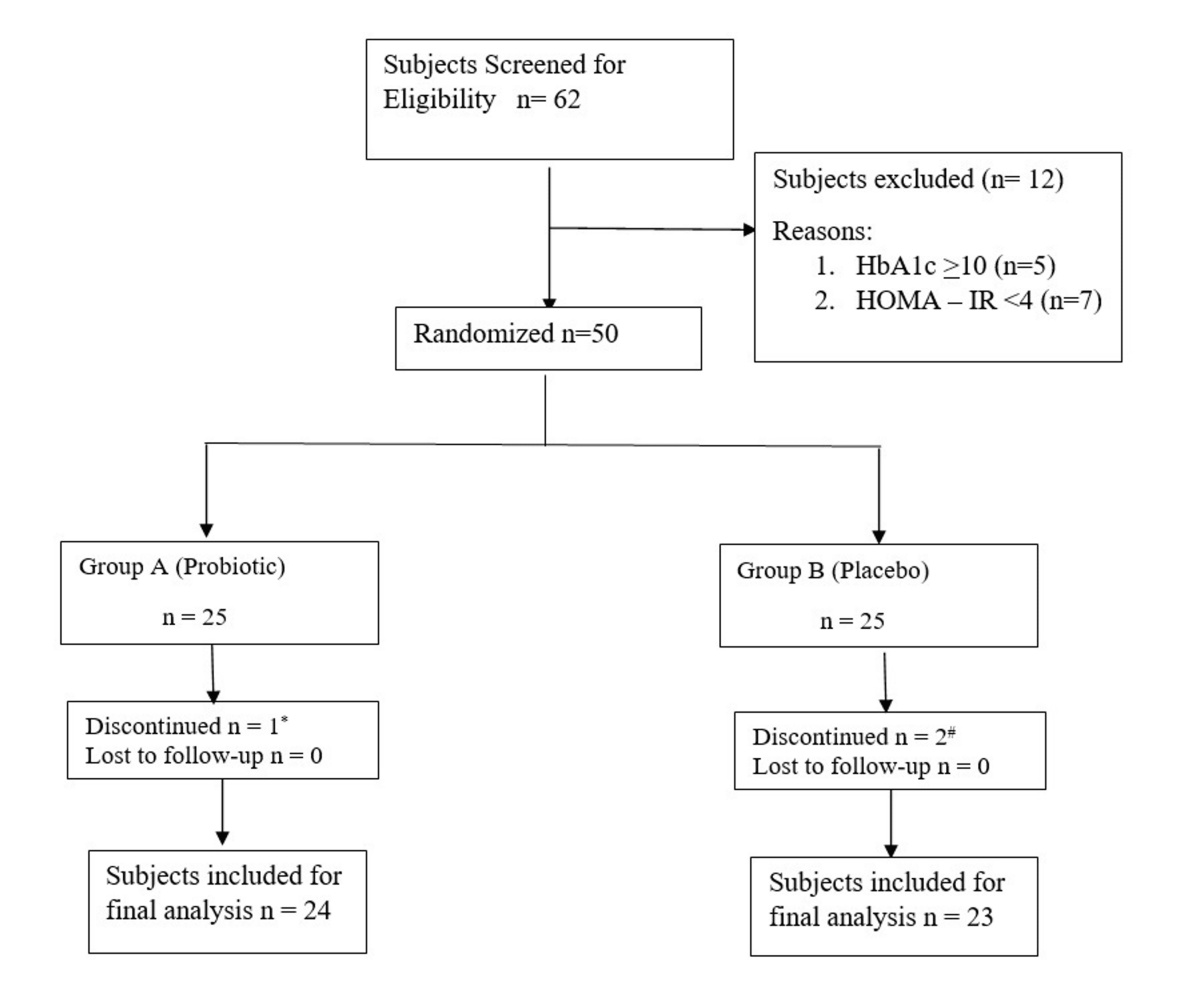
Participant flow diagram ^*^Discontinued due to change in location; ^#^discontinued due to personal reasons

Sixty-two participants were initially screened; however, only 50 met the eligibility criteria and were enrolled in the study. Twelve subjects were excluded because they did not meet the specific criteria. Of the 50 enrolled subjects, 47 were included in the final analysis because of discontinuation. The participant flow diagram is depicted in Figure 1.

Of the 50 enrolled subjects, 32 were men and 18 were women, with a mean age of 52.68 ± 6.75 years. The demographic and baseline characteristics of the study population are presented in Table 1. Most of the study variables were normally distributed, as determined by the Shapiro-Wilk test and Kolmogorov-Smirnov test.

**Table 1:** Baseline characteristics M: male; F: female; DM: diabetes mellitus; FBS: fasting blood sugar; HbA1c: hemoglobin A1c; SFI: serum fasting insulin: HOMA-IR: homeostatic model assessment for insulin resistance; TC: total cholesterol; HDL: high-density lipoprotein; TG: triglyceride; LDL: low-density lipoprotein; VLDL-C: very-low-density lipoprotein cholesterol; MDA: malondialdehyde; NO: nitric oxide; GSH: glutathione

Parameter	Group A (n = 24)	Group B (n = 23)	p-value
Age (years)	53.24	52.12	0.5
Height (cm)	158	162	0.4
Gender (M:F)	14:10	16:07	
Weight (kg)	62.4	66.2	0.1
BMI (kg/m^2^)	24.86	25.1	0.5
Duration of DM (years)	13.5 ± 2.25	12.83 ± 2.33	0.31
Years on dialysis	2.27 ± 0.91	2.17 ± 1.16	0.75
FBS (mg/dL)	114.29 ± 13.78	115.43 ± 8.18	0.73
HbA1c (%)	8.39 ± 0.6	8.37 ± 0.62	0.89
SFI (µIU/mL)	18.96 ± 2.11	19.03 ± 2.79	0.91
HOMA-IR	5.39 ± 0.89	5.47 ± 0.77	0.75
TC (mg/dL)	159 ± 35.44	153 ± 25.31	0.51
HDL (mg/dL)	35.17 ± 6.48	37.52 ± 7.79	0.26
TG (mg/dL)	143.38 ± 18.22	147.96 ± 15.91	0.36
LDL (mg/dL)	95.16 ± 35.99	85.97 ± 28.52	0.33
VLDL-C (mg/dL)	28.68 ± 3.64	29.59 ± 3.18	0.36
MDA (µmol/L)	5.28 ± 1.29	5.39 ± 1.35	0.76
NO (µmol/L)	64.06 ± 25.79	64.04 ± 20.88	0.99
GSH (µmol/L)	492.5 ± 45.63	490.66 ± 20.88	0.86

In this randomized double-blind placebo-controlled study to assess the efficacy and safety of probiotics in diabetic patients with ESRD on maintenance HD, we observed a significant reduction in the IR measured by HOMA-IR in group A (probiotic) from baseline to 12 weeks, as well as a significant decrease in HOMA-IR values in group A compared to group B (placebo) at the end of 12 weeks of treatment. Changes in OS biomarkers showed a significant reduction in MDA and improvement in NO and GSH levels in the probiotic group at 12 weeks compared to the baseline values. However, no such changes were observed in the placebo group. There was a significant difference in MDA and GSH values at the end of 12 weeks in the probiotic group compared to those in the placebo group. There was an improvement in NO levels in the probiotic group at the end of 12 weeks when compared to the placebo group; however, the difference was not statistically significant (p = 0.068). The changes in primary endpoints are represented in Table 2.

**Table 2:** Changes in primary outcome parameters SD: standard deviation; HOMA-IR: homeostatic model assessment for insulin resistance; MDA: malondialdehyde; NO: nitric oxide; GSH: glutathione

Characteristic	Group A (n = 24) mean ± SD		Within-group comparison (group A) (12 weeks - 0 weeks)		Group B (n = 23) mean ± SD		Within-group comparison (group B) (12 weeks - 0 weeks)		Between-group comparison (12 weeks) (A - B)	
	0 weeks	12 weeks	Mean difference	p-value	0 weeks	12 weeks	Mean difference	p-value	Mean difference	p-value
HOMA-IR	5.39 ± 0.91	4.73 ± 0.89	-0.66	0.006	5.47 ± 0.77	5.33 ± 0.83	0.07	0.71	-0.81	0.002
MDA (μM/L)	5.28 ± 1.29	4.36 ± 0.65	-0.92	0.001	5.39 ± 1.35	5.35 ± 0.61	-0.05	0.88	-0.99	0.001
NO (μM/L)	64.06 ± 25.79	70.22 ± 19.98	6.16	0.004	64.04 ± 20.88	60.21 ± 16.47	-3.83	0.07	10.01	0.068
GSH (μM/L)	492.5 ± 45.63	516.88 ± 45.19	24.37	0.001	490.66 ± 20.88	495.07 ± 24.09	4.40	0.21	21.81	0.046

Glycemic control assessed by FBS, serum fasting insulin, and HbA1c was also significantly improved in the probiotic group at 12 weeks when compared to the baseline values. No improvement was observed in the placebo group. The between-group analysis at 12 weeks showed a significant improvement in FBS, serum fasting insulin, and HbA1c values in the probiotic group when compared to the placebo group.

It was observed that there was improvement in TC, HDL-C, and LDL-C at 12 weeks in the probiotic group when compared to baseline; however, these improvements were not statistically significant. No improvement in the TG and VLDL-C values was noted at 12 weeks in the probiotic group compared to the baseline values. The changes in the lipid profile in the placebo group at 12 weeks compared to baseline did not show any improvement. Between-group analysis did not show significant differences in lipid parameters in the groups at the end of 12 weeks. The changes in secondary outcome measures are represented in Table 3.

**Table 3:** Changes in secondary outcome parameters SD: standard deviation; HOMA-IR: homeostatic model assessment for insulin resistance; FBS: fasting blood sugar; SFI: serum fasting insulin; HbA1c: hemoglobin A1c; LDL-C: low-density lipoprotein cholesterol; HDL-C: high-density lipoprotein cholesterol; VLDL-C: very-low-density lipoprotein cholesterol; TG: triglyceride; TC: total cholesterol

Characteristic	Group A (n = 24) mean ± SD		Within-group comparison (group A) (12 weeks - 0 weeks)		Group B (n = 23) mean ± SD		Within-group comparison (group B) (12 weeks - 0 weeks)		Between-group comparison (12 weeks) (A - B)	
	0 weeks	12 weeks	Mean difference	p-value	0 weeks	12 weeks	Mean difference	p-value	Mean difference	p-value
FBS (mg/dL)	114.29 ± 13.78	107.29 ± 11.53	-7.00	0.02	115.43 ± 8.18	116.26 ± 9.44	0.83	0.68	-8.97	0.005
SFI (µIU/mL)	18.96 ± 2.11	17.78 ± 2.1	-1.18	0.01	19.03 ± 2.79	19.17 ± 2.69	0.14	0.74	-1.39	0.054
HbA1c (%)	8.39 ± 0.6	8.06 ± 0.54	-0.33	0.003	8.37 ± 0.62	8.40 ± 0.50	0.03	0.78	-0.34	0.03
LDL-C (mg/dL)	95.16 ± 35.99	89.59 ± 31.45	-5.60	0.30	85.97 ± 28.52	83.7 ± 25.38	2.27	0.69	5.85	0.487
HDL-C (mg/dL)	35.17 ± 6.48	36.71 ± 4.21	1.54	0.16	37.52 ± 7.79	37.83 ± 5.8	0.30	0.80	-1.12	0.451
VLDL-C (mg/dL)	28.68 ± 3.64	29.32 ± 4	0.64	0.49	29.59 ± 3.18	29.77 ± 3.13	0.18	0.83	0.46	0.66
TG (mg/dL)	143.38 ± 18.22	146.58 ± 20	3.21	0.49	147.96 ± 15.91	148.87 ± 15.67	0.91	0.83	-2.29	0.665
TC (mg/dL)	159 ± 35.44	155.58 ± 28.32	-3.42	0.54	153 ± 25.31	151.30 ± 23.45	-1.78	0.74	4.28	0.576

Safety assessment

Five adverse events were noted in the 50 enrolled patients. No serious adverse drug events were observed during the 12 weeks of the study. The most common adverse event reported was gastrointestinal symptoms, which were mild and self-limiting in both study groups. Table 4 shows the adverse events in the study groups.

**Table 4:** Incidence of adverse events

Adverse events	Group A (probiotic) (n = 25)	Group B (placebo) (n = 25)
Bloating	2	0
Nausea	0	1
Constipation	1	1
Total	3	2

## Discussion

The present study was conducted to evaluate the effects of probiotics on IR, OS markers, glycemic control, and lipid profile in patients with diabetes and ESRD undergoing maintenance HD. In our randomized double-blind placebo-controlled study, it was observed that treatment with the probiotic UB0316 capsule (*Lactobacillus salivarius*, *Lactobacillus casei*, *Lactobacillus plantarum*, *Lactobacillus acidophilus*, *Bifidobacterium breve*, and *Bacillus coagulans* and fructooligosaccharide 100 mg) 30 billion CFU once a day for 12 weeks improved IR, with a mean difference of 0.66 in the HOMA-IR value, which was statistically significant (p = 0.006) in the probiotic group. In the placebo group, no such reduction was observed at 12 weeks compared to baseline.

There has been prior evidence of a decrease in IR among SCFA-producing bacteria in humans [18]. In our study, SCFA-producing strains of *Lactobacillus*, *Bifidobacterium*, and *Bacillus* were supplemented, which suggests a possible explanation for the lower HOMA-IR levels in the probiotic group.

A study conducted by Soleimani et al. [17] assessed the effect of regular HD on IR. The intervention was a probiotic containing *Lactobacillus acidophilus*, *Lactobacillus casei*, and *Bifidobacterium bifidum* (2 × 10^9^ CFU/g) for 12 weeks. They observed a significant reduction in HOMA-IR values with a mean difference of 2.9 in the probiotic group after 12 weeks of therapy.

A synbiotic (*Lactobacillus acidophilus*, *Lactobacillus casei*, and *Bifidobacterium bifidum* (2 × 10^9^ CFU/day each) (probiotic) + 0.8 g/day of inulin (prebiotic)) was given to diabetic patients with ESRD on maintenance HD for 12 weeks in another study by Soleimani et al. [19]. This study also demonstrated a significant decrease in IR with a mean difference of 2.5 in HOMA-IR values in the synbiotic group after 12 weeks of treatment.

Twelve weeks of probiotic supplementation in our study significantly increased the levels of GSH and NO. MDA levels were also lowered. At 12 weeks, the probiotic group in the Soleimani et al. trial showed a substantial reduction in MDA levels; however, the changes in NO and GSH levels were not statistically significant. Comparable outcomes were seen in the synbiotic study of Soleimani et al. [19] for markers of OS.

After 12 weeks of probiotic treatment, the effects of probiotics on the glycemic control indicators FBS, serum fasting insulin, and HbA1c were significantly reduced. Comparing the results to the research by Soleimani et al. [17], which shows a substantial decrease in FBS, serum fasting insulin, and HbA1c, revealed improvement in glycemic control. Comparable results were seen in the synbiotic experiment by Soleimani et al. [19] regarding glucose control.

The changes in the lipid profile in our study did not show any significant improvement in lipid parameters, which is consistent with the works on probiotics and synbiotics in HD patients with diabetes [17,19], in which the parameters for the lipid profile did not show any meaningful improvement. It is important to note that these studies were conducted over a period of 12 weeks, and longer-term studies may be needed to fully understand the effects of probiotics on the lipid profiles in this population.

A study conducted by Kooshki et al. [20] evaluated the effects of administering synbiotic (100 mg of lactol probiotic, which contains *Lactobacillus coagulans* and fructooligosaccharides (prebiotic)) supplements daily for eight weeks on serum inflammation, OS markers, and lipid profile in HD patients, demonstrating that synbiotic supplementation reduced serum inflammation and OS markers, as well as TC and LDL-C levels.

A randomized double-blind placebo-controlled trial conducted by Eslamparast et al. [21] showed that 28-week synbiotic supplementation with 2 × 10^8^ CFU/day of seven strains of bacteria (*Lactobacillus casei*, *Lactobacillus rhamnosus*, *Streptococcus thermophilus*, *Bifidobacterium breve*, *Lactobacillus acidophilus*, *Bifidobacterium longum*, and *Lactobacillus bulgaricus*) and prebiotics (250 mg fructooligosaccharide) significantly improved IR and glucose homeostasis in patients with metabolic syndrome.

Probiotic supplementation for 12 weeks improved glycemic control, OS indicators including MDA and GSH, and the lipid panel in diabetic nephropathy patients, according to a randomized double-blind placebo-controlled study conducted by Sadeghi et al. [22]. This suggests that probiotic intake may improve glycemic control and the favorable effects on oxidative damage by producing SCFA in the gut and decreasing the production of hydrogen peroxide radicals reducing lipid peroxidation.

Probiotic supplementation in diabetic nephropathy patients significantly reduced IR, glycemic control, and MDA, according to a systematic review and meta-analysis conducted by AbdelQadir et al. [23] for the efficacy of probiotics. However, no significant improvement in lipid profile or other OS markers (NO, GSH) was noted.

A systematic review and meta-analysis by Tao et al. [24] evaluated the effectiveness of probiotics in T2DM by analyzing 15 randomized controlled trials with a total of 902 participants. This study found that probiotic supplements may reduce HbA1c, FBS, and IR levels in T2DM patients. The results were verified through sensitivity analysis, which confirmed the reliability and stability of the findings.

The medications used in this study were well tolerated. No serious adverse events were reported. Mild gastrointestinal adverse effects were observed in this study. None of the subjects discontinued the study owing to adverse events, which suggests a good safety profile of the probiotic used in this study. The safety profile of probiotics in different populations including diabetic patients with ESRD on HD showed that probiotics were well tolerated, and no serious adverse effects were noted with the probiotic supplementations in studied populations [17,19-24].

Strengths and limitations

This is a first-of-kind study of the Indian population and explores a novel theoretical hypothesis; it can potentially lead to a deeper understanding of the topic. The current study assessed the outcomes for a short duration (12 weeks); studies to assess the long-term benefits of probiotics in this population would be helpful in establishing a potential clinical benefit and determining the long-term safety of probiotics. Another limitation is we did not assess direct dynamic measures like the hyperinsulinemic clamp or the glucose tolerance test. Consequently, this needs to be considered while interpreting our results.

## Conclusions

The present study indicated that probiotic supplementation for 12 weeks among diabetic ESRD patients on maintenance HD had beneficial effects on the parameters of glucose homeostasis and biomarkers of OS and was well tolerated. According to our study, probiotic supplementation may be an important and beneficial therapeutic approach for diabetic patients undergoing HD and can be recommended as an adjuvant to the standard of care. However, long-term studies are advised to confirm the positive clinical benefits of probiotic supplementation in patients undergoing HD.
